# Automated Cluster Detection of Health Care–Associated Infection Based on the Multisource Surveillance of Process Data in the Area Network: Retrospective Study of Algorithm Development and Validation

**DOI:** 10.2196/16901

**Published:** 2020-10-23

**Authors:** Yunzhou Fan, Yanyan Wu, Xiongjing Cao, Junning Zou, Ming Zhu, Di Dai, Lin Lu, Xiaoxv Yin, Lijuan Xiong

**Affiliations:** 1 Department of Nosocomial Infection Management, Union Hospital Tongji Medical College Huazhong University of Science and Technology Wuhan China; 2 School of Public Health Tongji Medical College Huazhong University of Science and Technology Wuhan China

**Keywords:** health care–associated infection, cluster detection, early warning, multi sources surveillance, process data

## Abstract

**Background:**

The cluster detection of health care–associated infections (HAIs) is crucial for identifying HAI outbreaks in the early stages.

**Objective:**

We aimed to verify whether multisource surveillance based on the process data in an area network can be effective in detecting HAI clusters.

**Methods:**

We retrospectively analyzed the incidence of HAIs and 3 indicators of process data relative to infection, namely, antibiotic utilization rate in combination, inspection rate of bacterial specimens, and positive rate of bacterial specimens, from 4 independent high-risk units in a tertiary hospital in China. We utilized the Shewhart warning model to detect the peaks of the time-series data. Subsequently, we designed 5 surveillance strategies based on the process data for the HAI cluster detection: (1) antibiotic utilization rate in combination only, (2) inspection rate of bacterial specimens only, (3) positive rate of bacterial specimens only, (4) antibiotic utilization rate in combination + inspection rate of bacterial specimens + positive rate of bacterial specimens in parallel, and (5) antibiotic utilization rate in combination + inspection rate of bacterial specimens + positive rate of bacterial specimens in series. We used the receiver operating characteristic (ROC) curve and Youden index to evaluate the warning performance of these surveillance strategies for the detection of HAI clusters.

**Results:**

The ROC curves of the 5 surveillance strategies were located above the standard line, and the area under the curve of the ROC was larger in the parallel strategy than in the series strategy and the single-indicator strategies. The optimal Youden indexes were 0.48 (95% CI 0.29-0.67) at a threshold of 1.5 in the antibiotic utilization rate in combination–only strategy, 0.49 (95% CI 0.45-0.53) at a threshold of 0.5 in the inspection rate of bacterial specimens–only strategy, 0.50 (95% CI 0.28-0.71) at a threshold of 1.1 in the positive rate of bacterial specimens–only strategy, 0.63 (95% CI 0.49-0.77) at a threshold of 2.6 in the parallel strategy, and 0.32 (95% CI 0.00-0.65) at a threshold of 0.0 in the series strategy. The warning performance of the parallel strategy was greater than that of the single-indicator strategies when the threshold exceeded 1.5.

**Conclusions:**

The multisource surveillance of process data in the area network is an effective method for the early detection of HAI clusters. The combination of multisource data and the threshold of the warning model are 2 important factors that influence the performance of the model.

## Introduction

Health care–associated infections (HAIs) are a socially sensitive and important public health issue that threatens patient safety, prolongs hospital stays, and increases economic burden. The incidence of HAIs in developed countries is 2%-6%, and in developing countries it is 12.6%-18.9% [1]. In China, the extra medical expenses per HAI patient varied from 9725 to 18,909 RMB (US $1427 to 2775) [2], and the total medical costs due to HAI have increased by nearly 70% [3]. Outbreaks are the main manifestation of the risk of HAIs, as HAIs are contagious, and approximately 2%-10% of HAI cases occur in the form of outbreaks [4]. In the past 40 years, there have been 465 major HAI outbreak events in China, with an average of 11.6 outbreak events annually reported by the media [5,6]. Because a significant number of HAI outbreaks have not been detected or reported in a timely manner, the severity of HAI outbreaks in China is likely to be seriously underestimated.

The key to establishing a methodology for HAI prevention and control is to develop a reliable outbreak warning system based on surveillance. To identify HAI outbreaks, HAI clusters must first be detected and then confirmed through epidemiological investigations. Therefore, detecting aggregated HAI cases is crucial to establishing a sound early warning system for HAI outbreaks. Traditional HAI surveillance is a form of passive monitoring, which relies on case reports by clinicians. However, owing to the compliance of clinicians with case reporting and the delay in HAI diagnosis, the timeliness of surveillance and warning for HAI outbreaks is limited.

In this paper, process data refer to the continuous, traceable, and basic information on patients who are admitted to hospitals; these data can be collected automatically by a search engine based on the local area network of the hospital. The proposed process data surveillance would be a form of active monitoring, which would not rely on delayed case reports. Therefore, the use of infection-related process data to detect the aggregation of HAI cases is likely to be a reliable method of early warning for HAI outbreaks. In recent years, the rapid development of information technology has led to a noticeable improvement in process data collection. Consequently, automated surveillance using process data related to infections has become a widely researched topic among the researchers of early warning systems for HAI outbreaks.

Recent studies have used a large amount of process data related to infections to identify HAI clusters [7-12]. However, surveillance that relied on a single indicator of process data limited the accuracy of HAI cluster detection because a solo process indicator was not sufficiently specific to reflect the occurrence and progress of infections. Some studies have confirmed that multisource surveillance for health-related data could improve the accuracy and timeliness of outbreak warning for infectious diseases [13,14]. Therefore, we considered that if a variety of process indicators related to infections could be combined for surveillance, the accuracy of the detection of HAI clusters could also be improved.

In a previous study [15], we assessed the performance and feasibility of automated cluster detection of multidrug-resistant organism–related HAIs using data on antibiotic use. In this study, we conducted an integrated surveillance of 3 process indicators using an electronic records information system based on the local area network of the hospital, including the antibiotic utilization rate in combination, inspection rate of bacterial specimens, and positive rate of bacterial specimens. We then analyzed the different combinations of the warning signals of these multisource process surveillance data to verify their early warning capability for HAI cluster detection.

## Methods

### Study Design and Setting

This was a retrospective observational study. The time series data of HAI incidences and the 3 indicators of process data were collected from 4 HAI high-risk units in Wuhan Union Hospital (WHUH). WHUH is a tertiary hospital in Wuhan, China, with a 5000-bed capacity. The process data, in this study, included the antibiotic utilization rate in combination, inspection rate of bacterial specimens, and positive rate of bacterial specimens from the 4 units with the highest HAI incidences. All data presented are from January 1, 2017, to June 28, 2019. Indicators were collected weekly at the unit level.

Surveillance and demographic data are available in the Real-Time Nosocomial Infection Surveillance System (RT-NISS) database. Briefly, the RT-NISS is seamlessly connected with several electronic information systems, including the hospital information system, laboratory information system, and other information systems in the local area network of the hospital. The infection-related process data are extracted and stored in real time in the database. The details of the RT-NISS database have been previously described [16].

### Indicators of Process Data

All indicators in this study were obtained from the RT-NISS database. The process data associated with antibiotic use and bacterial culture were automatically extracted from data sets containing doctor’s advice and nursing records by the RT-NISS using web mining and web crawler technology. The 3 process data indicators in this study were calculated weekly within each unit.

The antibiotic utilization rate in combination was determined to be the proportion of the number of admitted patients who used more than 1 antibiotic (n) divided by the total number of admitted patients (N), that is, antibiotic utilization rate in combination = n/N × 100%; the inspection rate of bacterial specimens was calculated as the number of specimens that were collected for bacterial testing (i) divided by the number of admitted patients (N), that is, inspection rate of bacterial specimens = i/N; the positive rate of bacterial specimens was calculated as the number of positive specimens with cultured bacteria (p) divided by the number of specimens collected for bacterial testing (i), that is, positive rate of bacterial specimens = p/i × 100%.

**Figure 1:**
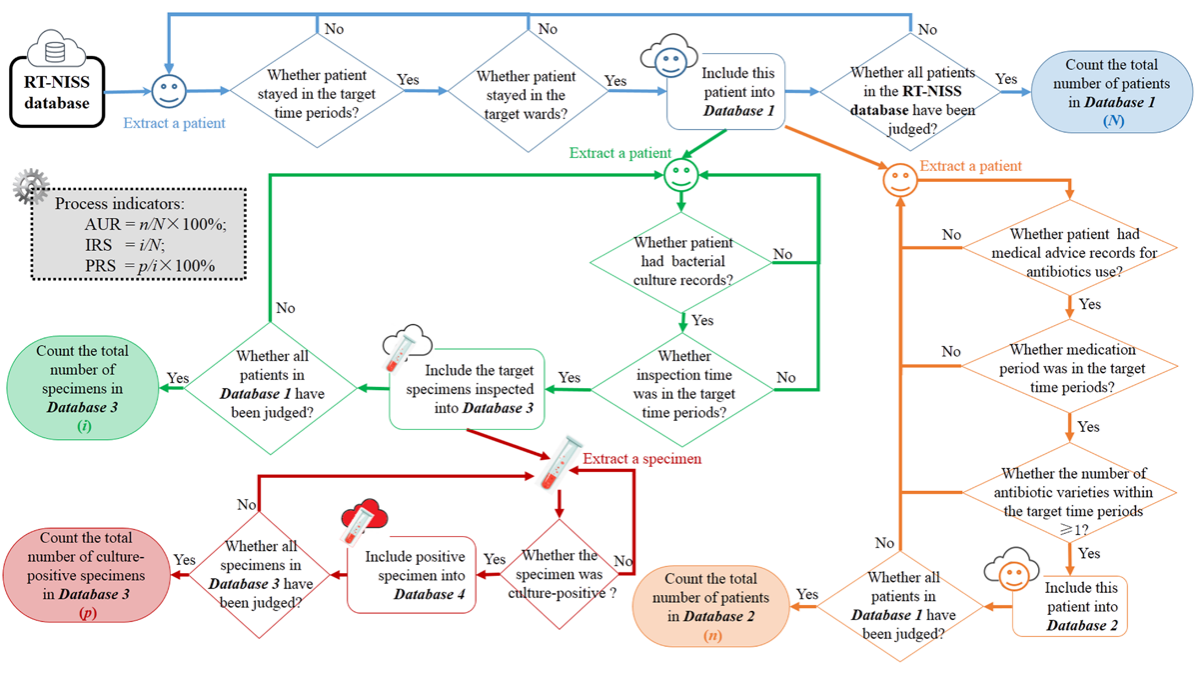
The flow diagram of data extraction process of the variables used to calculate the process indicators. RT-NISS: Real-time nosocomial infection surveillance system; AUR: Antibiotic utilization rate in combination; IRS: Inspection rate of bacterial specimens; PRS: Positive rate of bacterial specimens.

Data on the prescribed oral and intravenous antibiotics were collected, while topical antibiotics were excluded from the data collection. The sputum of bacterial culture included throat secretion, urine, blood, stool, pleural effusion, cerebrospinal fluid, ascites, and venous catheter, among others. Repeated samples from each individual were excluded. The data extraction process of the variables (N, n, i, and p) used to calculate the process indicators is shown in Figure 1.

### Identification of HAI Cases

HAI cases were identified according to the diagnostic criteria for HAIs, which were issued by the Ministry of Health of China in 2001 [17]. The HAI case findings were documented weekly by a hospital infection management team. The hospital infection management team comprised clinicians, nurses, and full-time infection control practitioners. All members within the hospital infection management team independently reviewed the clinical records of the patients to include reports of illness, microbiology data, antibiotic data, imaging reports, and results of clinical laboratory tests, and HAI cases were identified after the hospital infection management team members reached a consensus. The weekly HAI incidence was measured as the number of new HAI cases in a week divided by the total number of inpatients in that week.

### Warning Detection Model

In this study, the time series data sets of each surveillance indicator were analyzed using the Shewhart warning model, which is a common statistical process control for detecting clusters. We used a 4-week moving average of time series data in the Shewhart model, considering the inpatient’s average length of hospitalization and the epidemiologic characteristics of infected patients. We then used the data from the nearest 4 weeks before the current week as the dynamic warning baseline of the Shewhart model. Finally, the Shewhart warning statistics (S_t_) for each week were calculated using the mean and SD of the dynamic baseline data sets according to the following formula:

S_t_=(X_t_ – µ_t_)/σ_t_

where X_t_ is the observation value at week t; µ_t_ and σ_t_ are the mean and SD of the observation values for the warning baseline from week t–4 to week t–1, respectively. The warning signal at week t was generated when S_t_ exceeded the threshold.

An HAI cluster is considered to exist when a group of HAIs occurs closely together in a health care unit, so the previous warning threshold of an HAI cluster was based on the statistical variations in the frequency. The Shewhart model with a threshold of 2.0 was used for detecting HAI clusters in WHUH according to the Guideline of Control of Health Care-Associated Infection Outbreak [18]. This implies that a warning signal for an HAI cluster was generated when the 4-week moving average of HAI incidence at the current week exceeded the mean plus 2 SDs of the past 4 weeks. We used 51 thresholds (0.0-5.0, steps of 0.1) to detect process data clusters to explore the optimal threshold of the Shewhart warning model for process data warning.

### Warning Strategies for Process Data

We designed 5 warning strategies of process indicators based on the combination of 3 single-indicator warning strategies: (1) antibiotic utilization rate in combination only, (2) inspection rate of bacterial specimens only, and (3) positive rate of bacterial specimens only, and 2 multi-indicator warning strategies, (4) antibiotic utilization rate in combination + inspection rate of bacterial specimens + positive rate of bacterial specimens in parallel, and (5) antibiotic utilization rate in combination + inspection rate of bacterial specimens + positive rate of bacterial specimens in series. The parallel warning signal is generated once any subindicator generates a signal, and the series warning signal is generated only when all subindicators generate signals during the same period.

### Comparison of Warning Signals of Process Data With HAI Incidence

We used the consistency of warning signals between the HAI incidence and process data to evaluate the warning performance for HAI cluster detection. The warning signals of the process data were considered as the test and those of the HAI incidences as references. The early warning signal was defined as the signal of process data generated earlier than the signal of HAI incidence within the 4-week period. Accordingly, we calculated the sensitivity, specificity, and Youden index under each threshold of process data for the early detection of HAI clusters. Furthermore, the receiver operating characteristic (ROC) curve of the process data for the early detection of the signals of HAI clusters was plotted using sensitivity and 1–specificity under 51 thresholds (0.0 to 5.0, steps of 0.1). Youden index was used to evaluate the comprehensive warning performance for HAI cluster detection under each threshold.

Sensitivity = Number of HAI cluster signals detected by the early warning signals/Total number of HAI cluster signals

Specificity = Number of weeks that signal generated neither in HAI incidence nor in process indicators/Number of weeks that no signal generated in the HAI incidence

Youden index = Sensitivity + Specificity–1

### Statistical Analysis

The one-way analysis of variance was used to compare the differences between the mean values, and a chi-square test was used to compare the differences between the proportions among the 4 independent units. A statistical evaluation of Youden index among the warning strategies in each threshold was performed using the paired samples *t* test. A *P*-value of .05 or less was considered statistically significant in all analyses.

## Results

### Demographic Characteristics

A total of 23,119 patients were admitted to the 4 HAI high-risk units in WHUH during the study period. The hospital infection management team diagnosed 1503 HAI cases. The HAI incidence in these high-risk units ranged from 5.36% (462/8618 patients) to 9.06% (316/3489 patients). Statistically significant differences were observed in all demographic characteristics of patients among the 4 HAI high-risk units (Table 1).

**Table 1:** Demographic characteristics of inpatients in the 4 high-risk units in Wuhan Union Hospital during the surveillance period.

Characteristics		Total	High-risk unit				*P* value
			Unit 1	Unit 2	Unit 3	Unit 4	
**Participants (N)**		23,119	8618	7414	3598	3489	
	Male, n (%)	13,153 (56.9)	4679 (54.3)	4284 (57.8)	2117 (58.8)	2073 (59.4)	<.001
	Age in years, mean (SD)	44.9 (21.7)	47.3 (16.3)	34.1 (25.9)	51.1 (15.1)	55.6 (19.5)	<.001
	Hospitalization days, mean (SD)	19.6 (26.2)	15.2 (32.3)	24.1 (15.5)	16.1 (13.1)	24.6 (34.1)	<.001
	Surgical procedure, n (%)	13,747 (59.5)	3459 (40.1)	6386 (86.1)	1503 (41.8)	2399 (68.8)	<.001
	Mechanical ventilation, n (%)	10,496 (45.4)	371 (4.3)	6828 (92.1)	227 (6.3)	3077 (88.2)	<.001
	Central venous catheter, n (%)	8485 (36.7)	353 (4.1)	6643 (89.6)	450 (12.5)	1026 (29.4)	<.001
	Urinary catheter, n (%)	17,779 (76.9)	5378 (62.4)	7051 (95.1)	1979 (55.0)	3370 (96.5)	<.001
	Health care–associated infection, n (%)	1503 (6.5)	462 (5.4)	418 (5.6)	307 (8.5)	316 (9.1)	<.001
	Antibiotics used, n (%)	18,124 (78.4)	4736 (55.0)	7214 (97.3)	2749 (76.4)	3425 (98.2)	<.001
	Antibiotic days, mean (SD)	10.7 (11.3)	5.6 (8.7)	13.2 (8.6)	10.9 (12.1)	17.6 (15.1)	<.001
	Antibiotics used in combination, n (%)	6356 (27.5)	1010 (11.7)	1895 (25.6)	1166 (32.4)	2285 (65.5)	<.001
	Antibiotic days in combination used, mean (SD)	2.7 (6.6)	1.0 (3.5)	2.2 (5.2)	3.6 (8.1)	7.2 (10.3)	<.001
	Microbiological test, n (%)	6040 (26.1)	1415 (16.4)	1596 (21.5)	1262 (35.1)	1767 (50.6)	<.001
	Microbiological test with positive result, n (%)	3129 (13.5)	728 (8.4)	677 (9.1)	632 (17.6)	1092 (31.3)	<.001
**Microbiological specimens**		43,070	11,785	8685	5647	16,953	
	Positive, n (%)	10,086 (23.4)	2600 (22.1)	1551 (17.9)	1927 (34.1)	4008 (23.6)	<.001
**Isolated strains**		11,808	3070	1679	2326	4733	
	*Acinetobacter baumannii*, n (%)	3430 (29.0)	769 (25.0)	456 (27.2)	319 (13.7)	1886 (39.8)	<.001
	*Staphylococcus aureus*, n (%)	1683 (14.3)	581 (18.9)	88 (5.2)	535 (23.0)	479 (10.1)	<.001
	*Pseudomonas aeruginosa*, n (%)	1214 (10.3)	362 (11.8)	219 (13.0)	126 (5.4)	507 (10.7)	<.001
	*Klebsiella pneumonia*, n (%)	1076 (9.1)	326 (10.6)	166 (9.9)	322 (13.8)	262 (5.5)	<.001
	*Saccharomyces albicans*, n (%)	792 (6.7)	148 (4.8)	159 (9.5)	176 (7.6)	309 (6.5)	<.001
	*Escherichia coli*, n (%)	624 (5.3)	119 (3.9)	78 (4.6)	223 (9.6)	204 (4.3)	<.001
	Other, n (%)	2989 (25.3)	765 (24.9)	513 (30.6)	625 (26.9)	1086 (22.9)	<.001

### Surveillance and Cluster Detection

**Figure 2:**
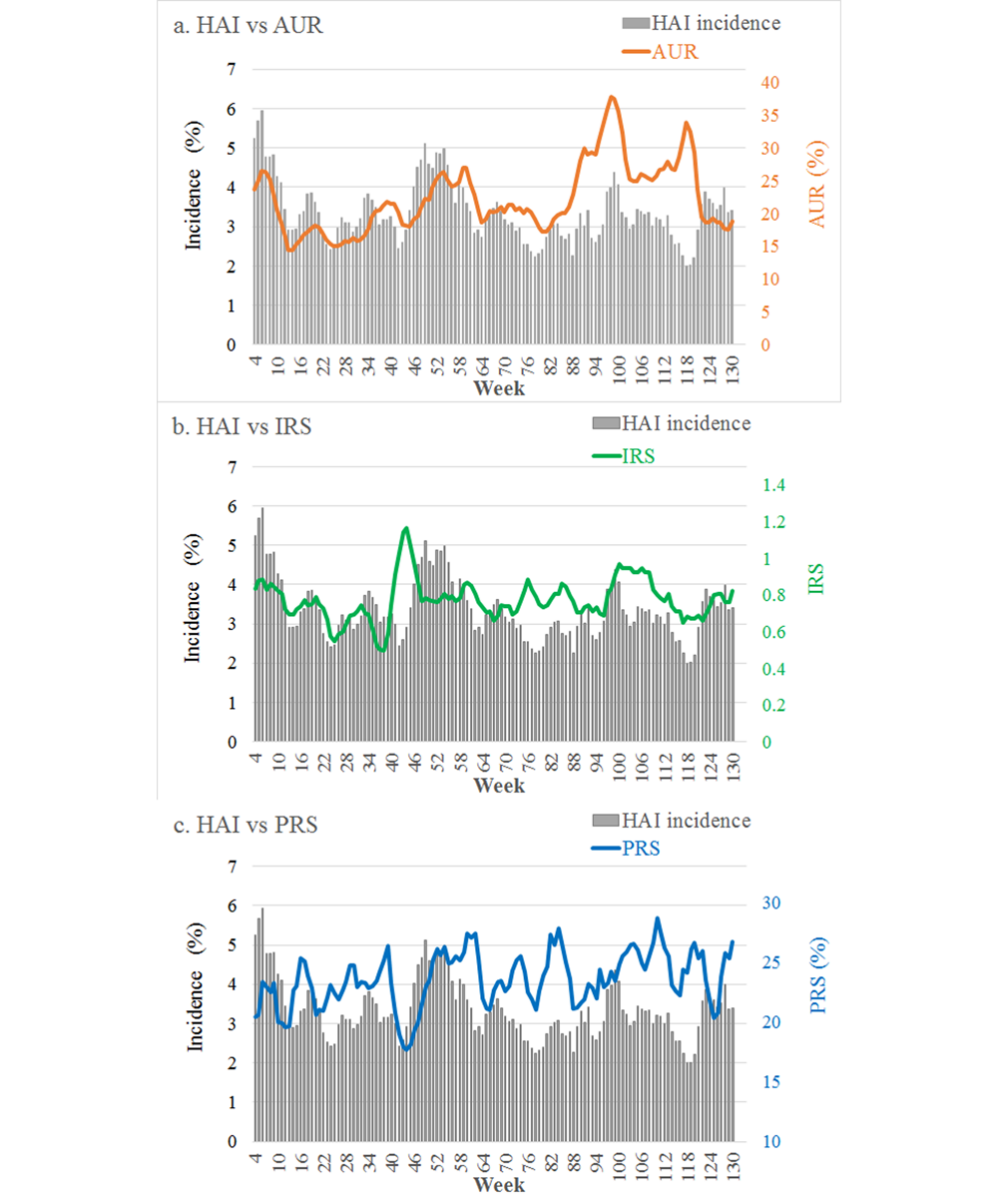
The time-series charts comparison of process data with HAI incidence in all surveillance units. AUR: Antibiotic utilization rate in combination; IRS: Inspection rate of bacterial specimens; PRS: Positive rate of bacterial specimens.

The time series charts of the 3 process indicators and HAI incidences for all units are shown in Figure 2, as well as in Multimedia Appendix 1. The fluctuations of the time series in the process data are generally synchronous with those in HAI incidence. For the HAI cluster detection using the Shewhart warning model in each unit, there were 20 signals generated in unit 1, 16 signals in unit 2, 18 signals in unit 3, and 16 signals in unit 4. These HAI cluster signals were compared with those of the process data warning at each threshold. An example of signal comparison at the threshold of 2.0 is shown in Multimedia Appendix 1.

### Warning Detection Evaluation

**Figure 3:**
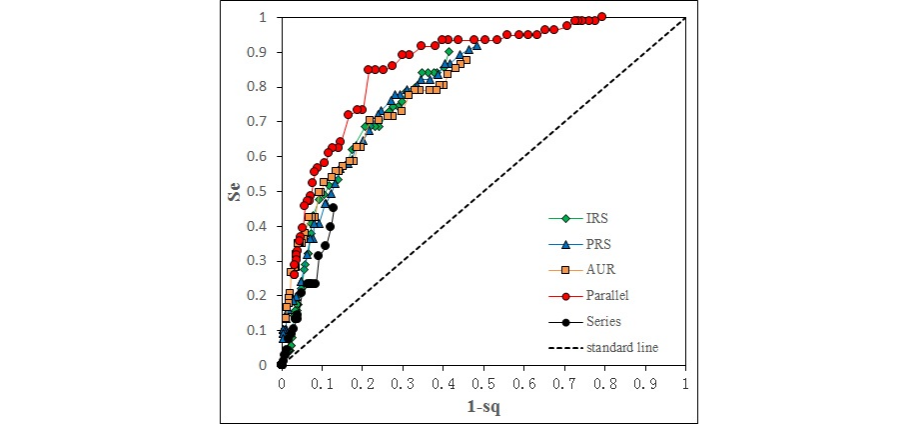
The ROCs of five warning strategies of process data for identifying signals of HAI clusters. Fifty-one thresholds (0.0 to 5.0 step by 0.1) were used for detecting clusters of process data. Dots indicate the sensitivities and 1-specificities for each threshold. AUR: Antibiotic utilization rate in combination; IRS: Inspection rate of bacterial specimens; PRS: Positive rate of bacterial specimens.

According to the definition of early warning signals, the ROC curves of 5 warning strategies for early detected HAI cluster signals were plotted using scattered points of 51 thresholds. Figure 3 depicts the overall ROC curves of process data warning for detecting HAI cluster signals across the 4 units. Generally, all ROC curves are located above the standard line, and the area under the ROC curve is larger in the parallel warning strategy than in the single-indicator warning strategies and the series warning strategy.

The optimal Youden index for the early detection of HAI cluster signals was higher in the parallel warning strategy than in any other warning strategies. Specifically, the optimal Youden indexes were 0.48 (95% CI 0.29-0.67) at a threshold of 1.5 for antibiotic utilization rate in combination only, 0.49 (95% CI 0.45-0.53) at a threshold of 0.5 for inspection rate of bacterial specimens only, 0.50 (95% CI 0.28-0.71) at a threshold of 1.1 for positive rate of bacterial specimens only, 0.63 (95% CI 0.49-0.77) at a threshold of 2.6 in the parallel strategy, and 0.32 (95% CI 0.00-0.65) at a threshold of 0.0 in the series strategy.

**Figure 4:**
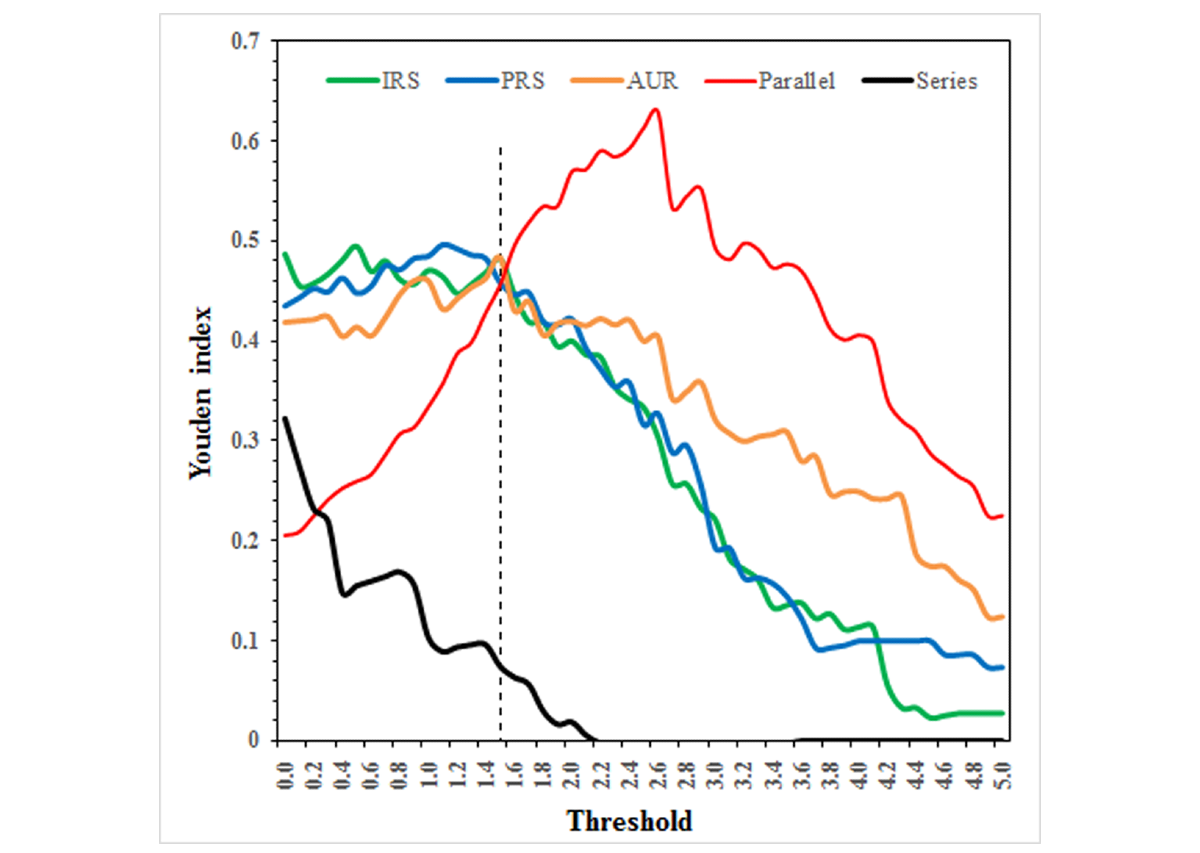
The curves of Youden index varied with thresholds of Shewhart detection model. AUR: Antibiotic utilization rate in combination; IRS: Inspection rate of bacterial specimens; PRS: Positive rate of bacterial specimens.

Figure 4 illustrates the overall curves of the Youden index variation with the warning thresholds across the 4 units. A threshold of 1.5 was the demarcation point of Youden index for judging the superiority between the parallel warning strategy and the single-indicator warning strategies.

Table 2 shows the mean difference in Youden index between the warning strategies. When the threshold of the Shewhart model was less than or equal to 1.5, Youden indexes in the single-indicator warning strategies were higher than those in the parallel warning strategy, and those of inspection rate of bacterial specimens only and positive rate of bacterial specimens only were better than that of antibiotic utilization rate in combination only; however, when the threshold was greater than 1.5, Youden indexes in the parallel warning strategy were higher than that in the single-indicator warning strategies, and Youden index of antibiotic utilization rate in combination only was better than those of inspection rate of bacterial specimens only and positive rate of bacterial specimens only. In addition, under most thresholds, Youden indexes in the series warning strategy were lower than those in the single-indicator warning strategies and parallel warning strategy.

**Table 2:** Threshold-matched comparison of Youden index of early warning detection for health care–associated infection clusters.

Threshold and comparison		Mean difference of Youden index (95% CI)	*t*	*df* (n–1)	*P* value
**Overall (from 0.0 to 5.0)**					
	IRS^a^ – PRS^b^	–0.011 (–0.023 to 0.001)	–1.877	203	.062
	IRS – AUR^c^	–0.062 (–0.085 to –0.038)	–5.206	203	<.001
	PRS – AUR	–0.051 (–0.072 to –0.030)	–4.797	203	<.001
	IRS – Parallel	–0.124 (–0.155 to –0.093)	–7.856	203	<.001
	PRS – Parallel	–0.112 (–0.142 to –0.083)	–7.450	203	<.001
	AUR – Parallel	–0.062 (–0.085 to –0.038)	–5.234	203	<.001
	IRS – Series	0.230 ( 0.206 to 0.254)	18.643	203	<.001
	PRS – Series	0.241 ( 0.218 to 0.264)	20.701	203	<.001
	AUR – Series	0.292 ( 0.273 to 0.311)	29.654	203	<.001
**Threshold ≤ 1.5 (from 0.0 to 1.5)**					
	IRS – PRS	0.002 (–0.019 to 0.023)	0.155	63	.878
	IRS – AUR	0.033 (0.001 to 0.065)	2.033	63	.046
	PRS – AUR	0.031 (0.008 to 0.054)	2.711	63	.009
	IRS – Parallel	0.161 (0.131 to 0.191)	10.646	63	<.001
	PRS – Parallel	0.159 (0.131 to 0.187)	11.217	63	<.001
	AUR – Parallel	0.128 (0.102 to 0.153)	10.037	63	<.001
	IRS – Series	0.309 (0.270 to 0.348)	15.851	63	<.001
	PRS – Series	0.308 (0.266 to 0.349)	14.750	63	<.001
	AUR – Series	0.276 (0.238 to 0.314)	14.489	63	<.001
**Threshold > 1.5 (from 1.6 to 5.0)**					
	IRS – PRS	–0.017 (–0.031 to –0.003)	–2.368	139	.019
	IRS – AUR	–0.105 (–0.133 to –0.077)	–7.388	139	<.001
	PRS – AUR	–0.088 (–0.115 to –0.062)	–6.614	139	<.001
	IRS – Parallel	–0.254 (–0.272 to –0.235)	–26.475	139	<.001
	PRS – Parallel	–0.237 (–0.255 to –0.218)	–25.011	139	<.001
	AUR – Parallel	–0.148 (–0.167 to –0.130)	–15.637	139	<.001
	IRS – Series	0.194 (0.165 to 0.223)	13.217	139	<.001
	PRS – Series	0.211 (0.184 to 0.237)	15.818	139	<.001
	AUR – Series	0.299 (0.277 to 0.322)	26.259	139	<.001

^a^IRS: inspection rate of bacterial specimens.

^b^PRS: positive rate of bacterial specimens.

^c^AUR: antibiotic utilization rate in combination.

## Discussion

### Principal Findings

In this study, we retrospectively analyzed the time series surveillance data in 4 HAI high-risk units in WHUH to evaluate the early warning performance of 3 process indicators (antibiotic utilization rate in combination, inspection rate of bacterial specimens, and positive rate of bacterial specimens) for detecting HAI clusters under different warning strategies. The ROC curves of all warning strategies are located above the standard line, indicating that surveillance based on process data was able to detect HAI clusters. Unit-specific results manifested similar outcomes in the 4 independent high-risk units, suggesting a universal warning capability of process data surveillance for HAI cluster detection. However, the accuracy of warnings varied in different units, mainly owing to the differences in population characteristics, antimicrobial utilization behaviors, and pathogenic spectrum.

Based on the correlation between process indicators and infections, process indicators have been used to detect HAI cases and outbreaks. In Freeman’s review of research progress in electronic HAI surveillance [19], 77% (34/44) of studies used electronic medical records to detect HAI cases. In another review of the automated detection of HAI outbreaks, 62% (18/29) of studies used microbiological data to detect HAI outbreaks [9]. For example, Fournier et al [10] demonstrated that the consumption of antibiotics for *Pseudomonas aeruginosa* infection could identify 3 epidemics of *P. aeruginosa* infections in a burn center [10]. Carron et al [20] suggested that the prospective electronic surveillance of drug consumption could identify the outbreaks of *P. aeruginosa* infections in the absence of routine traditional surveillance. Moreover, a recent retrospective study in the United States revealed that 9 HAI outbreaks between 2011 and 2016 were successfully detected via data mining of the electronic medical records database, and the earliest warning signal in one of the outbreaks could be generated when the second HAI patient was diagnosed [12]. In a study conducted in 2 hospitals in France, researchers used a space-time permutation scan statistics model to analyze the microbial data in the WHONET system and successfully detected several HAI outbreaks [11].

Combining multiple independent indicators together to detect HAI clusters would be a new research direction for the early warning of HAI outbreaks. Informatization technology provides a convenient tool for the real-time surveillance of multisource process data. Because process indicators are nonspecific for infections, monitoring a single indicator alone cannot fully reflect the occurrence and progression of an HAI, which may limit the accuracy and timeliness of HAI detection. To overcome this problem, a combination of multiple nonspecific indicators provides more infection-related information, which could be expected to improve the early warning performance of HAI detection. This hypothesis was confirmed in our study. The area under the ROC curve was higher for the multi-indicator parallel warning strategy than all other single-indicator warning strategies, indicating that the combined monitoring of multiple process indicators improves the performance of HAI cluster detection. Furthermore, other researchers have proposed similar views. Spolaore et al [21] suggested that the combination of multiple surveillance indicators improved the accuracy of surgical site infection detection. In their study, the positive predictive values for detecting surgical site infections using discharge codes alone or microbiology reports alone were only 70%, but the positive predictive value increased to 97% when these 2 indicators were used in combination.

It is worth mentioning that the combination of multiple indicators is an important factor that affects the accuracy of HAI cluster detection. In our study, compared with the single-indicator warning strategies, the area under the ROC curve was increased when using the parallel warning strategy but decreased when using the series warning strategy. The results of a Youden index comparison exhibited the same situation: the average value of Youden index under each threshold in the parallel warning strategy was greater than those in the single-indicator warning strategies, but the average value of Youden index under each threshold in the series warning strategy was lower than those in the single-indicator warning strategies. In general, the combination of multiple indicators in parallel could improve the sensitivity of warnings but decrease their specificity. Conversely, the combination of multiple indicators in series could improve the specificity of warnings but reduce their sensitivity. This situation was also examined by Bouzbid et al [22]. The sensitivity and specificity for HAI identification using the indicator of a drug prescriptions algorithm alone were 82.3% and 66.7%, respectively, and those using the indicator of the microbiological algorithm alone were 94.0% and 77.3%, respectively. Furthermore, when these 2 indicators were combined in parallel, the sensitivity increased to 99.3%, but the specificity decreased to 58.6%. When these 2 indicators were combined in series, the sensitivity reduced to 77.0%, and the specificity increased to 87.3%.

The threshold of the warning model is another important factor affecting the performance of HAI cluster detection. In prospective surveillance and warning, it was necessary to consider the risk severity and preventive costs of HAI clusters. The threshold of the warning model should be set according to the demand for warning sensitivity and the costs for responding to warning signals. From our results of the Youden index variation with the thresholds of the warning model in Figure 4, we found that when the threshold of the Shewhart model was 1.5 or less, the performance of the parallel warnings for HAI clusters was lower than that of the single-indicator warnings. Only when the threshold was greater than 1.5, the performance of the parallel warnings overtook the single-indicator warnings. Theoretically, a low threshold is prone to higher sensitivity and lower specificity for warnings, whereas a high threshold is prone to lower sensitivity and higher specificity. Owing to the opposing relationship between sensitivity and specificity, the maximum value of Youden index, which comprehensively considers sensitivity and specificity, could be regarded as an alternative criterion for determining the optimal threshold. Our results indicated that Youden index of parallel warnings was optimal at a threshold of 2.6. In addition, the optimal Youden index of parallel warnings exceeded that of single-indicator warnings; furthermore, the optimal Youden indexes of single-indicator warnings were higher than those of the series warnings. This result again proves that the parallel warning strategy could improve the performance of HAI cluster detection, while the series warning strategy reduced it.

Previous studies have reported some available novel methods for HAI outbreak detection, mainly including (1) exploration of new monitoring objects, (2) innovation of statistical models, and (3) application of intelligent algorithms.

A French project consortium confirmed the feasibility of natural language processing for automatic HAI detection in hospital facilities by developing a natural language processing solution for detecting HAI events in electronic medical records. The overall sensitivity and specificity of the automatic detection of HAIs were 83.9% and 84.2%, respectively [23]. This detection efficiency is similar to that of the multisource surveillance of process data in our study. Another study reported a novel statistical process control chart using Twitter’s anomaly and breakout algorithm to detect anomalous HAI surveillance data. It appeared to work better than the statistical process control charts in the context of seasonality and autocorrelation, showing an available algorithm for anomalous HAI detection [24]. In addition, Adhikari et al [25] introduced an efficient data- and model-driven algorithm to detect HAI outbreaks. They designed a near-optimal algorithm to obtain the monitoring data sets and simulated the spread of *Clostridium* *difficile* infection in hospitals. Their algorithm displayed a high sensitivity of 95% for HAI outbreak detection according to data simulation, better than many natural heuristics. In addition, researchers in the Ourense University Hospital Complex (Spain) developed the InNoCBR system for HAI surveillance based on the implementation of intelligent diagnosis for HAIs. Similar to our RT-NISS, the InNoCBR was established using databases of microbiology and pharmacy, but the difference is that it integrates an intelligent diagnostic module into the acquisition process module. The InNoCBR achieved a sensitivity of 70.83% and a specificity of 97.76%, displaying an acceptable detection performance for HAI surveillance [26]. In general, exploring high-quality monitoring data and an intelligent detection model would be the main direction of HAI detection in future research.

Some limitations regarding the generalizability of the findings in this study must be addressed. First, a false correlation likely exists in the warning signals between process data and HAI incidence. This study was a retrospective analysis based on historical surveillance data; thus, the correlation of warning signals between the process data and HAI incidence was judged according to the signal’s time and place, lacking epidemiological investigation. Therefore, the applicability of our results requires further research in prospective surveillance.

Second, the process indicators used in our study were a type of nonspecific data, which could provide limited information regarding the occurrence and progress of infections, so it is susceptible to generating negative signals when these nonspecific indicators are used to detect HAI clusters. Although the multiple indicators combined in parallel could improve the warning performance for detecting HAI clusters, they also increased the number of negative signals, resulting in excessive costs for responding to these false warning signals. Consequently, multisource surveillance based on process data could not completely replace the traditional case surveillance at present, and it would be an auxiliary method for detecting disease cases or clusters.

Finally, surveillance noise is an inevitable problem in the automatic surveillance systems based on process data. In fact, automated monitoring is a process of automatically retrieving, identifying, and collecting the formatted data from databases using computer technology. Although automatization improved surveillance efficiency, it was inevitable that some confounding information would be mixed into surveillance data. Because these confounding data, which add noise to surveillance, were usually stored in an unstructured form, it was difficult to automatically wash and refine them in our RT-NISS system. For example, the data on prophylactic medication and therapeutic medication for community infections were mixed into the indicator of antibiotic utilization rate in combination. In addition, some repeated cultures of blood specimens were mixed into the indicators of inspection rate of bacterial specimens and positive rate of bacterial specimens because blood specimens from adults were collected in 2-3 sets each time from different puncture points in WHUH, according to the Operating Procedures of Blood Culture for Clinical Microbiology Laboratory, as issued by the National Health Commission of China. Although these confounding noises could affect the performance of HAI cluster detection, we considered that manually washing and refining them was time-/labor-consuming, and this is contrary to the intention of automatic early warning. In fact, considering that infection control practitioners could investigate warnings more easily in the hospital than in the community, we suggest that it is acceptable to raise the timeliness of warnings at the expense of surveillance noises. We also believe that an automatic washing and refining function for these surveillance noises in HAI cluster detection will be achieved by artificial intelligence technology in the future.

### Conclusion

The multisource surveillance of process data in the area network could detect HAI clusters without relying on case reports; moreover, it has advantages in terms of timeliness and automation compared with traditional HAI case surveillance. In this study, we demonstrated that the automated monitoring of the process data of antibiotic utilization rate in combination, inspection rate of bacterial specimens, and positive rate of bacterial specimens could provide early warnings of HAI clusters. The combination of multiple indicators and the threshold of the detection model are 2 important factors affecting warning performance. Multiple data combined in parallel can improve the warning performance, whereas when combined in series, these data can reduce performance. A low threshold of the detection model is more suitable for the single-indicator warning strategies, whereas a high threshold is more suitable for multi-indicator warning strategies. Further prospective research is required to confirm the warning theory of multisource surveillance based on process data.

## Appendix

Multimedia Appendix 1 The unit-specific time-series charts of surveillance data and their warning signals (red dots) generated by Shewhart detection model at a threshold of 2.0.

## Abbreviations

HAI: Health care–associated infection
ROC: receiver operating characteristic
RT-NISS: Real-Time Nosocomial Infection Surveillance System
WHUH: Wuhan Union Hospital
