# Supplementary figures and images for: Automated Cluster Detection of Health Care–Associated Infection Based on the Multisource Surveillance of Process Data in the Area Network: Retrospective Study of Algorithm Development and Validation
Source: JMIR Med Inform. 2020 Oct 23;8(10):e16901. doi: 10.2196/16901 (PMC7647819; doi:10.2196/16901)

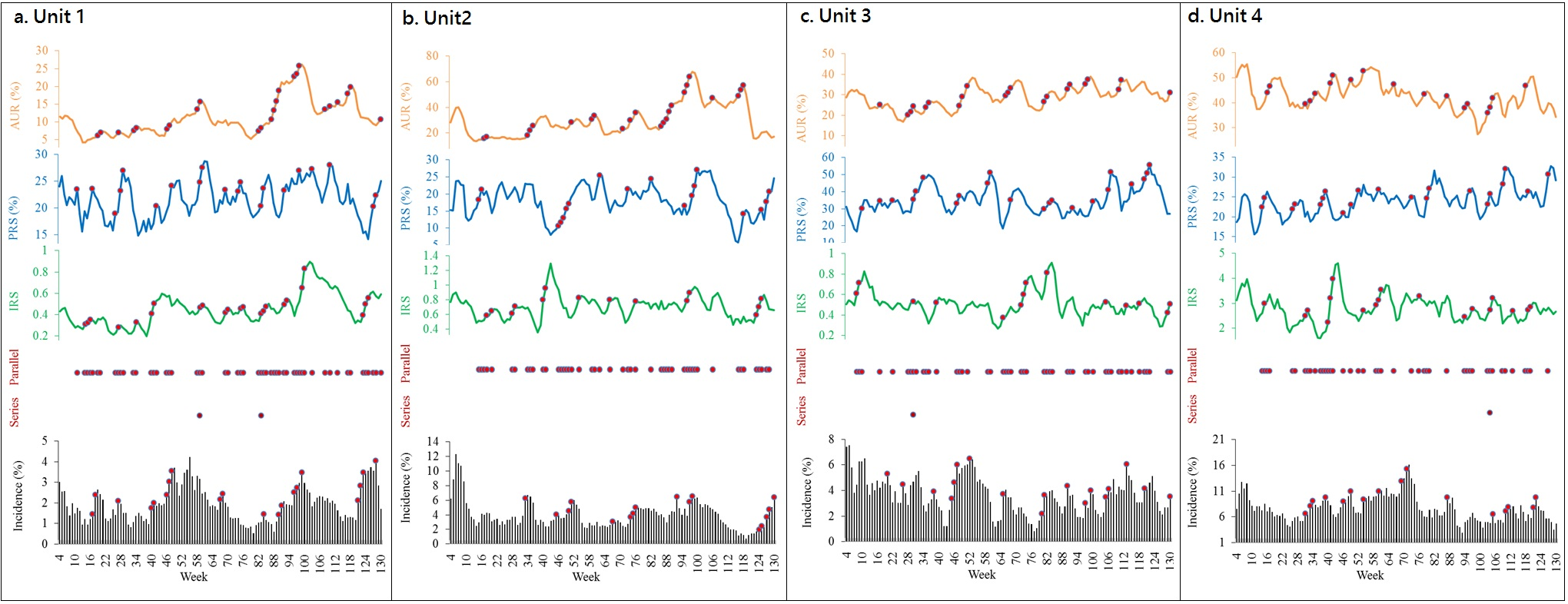

Supplement: Multimedia Appendix 1 [file medinform_v8i10e16901_app1.png]
